# Utilization of a Joint Point Regression Model for Predicting Mortality Rates of Common Cancers in Babol City

**DOI:** 10.1002/cnr2.70107

**Published:** 2025-01-22

**Authors:** Pouyan Ebrahimi, Sara Khaleghi, Mohebat Vali, Sahar Delavari, Soraya Khafri, Mohsen Karami, Layla Shojaie, Hossein‐Ali Nikbakht

**Affiliations:** ^1^ Student Research Committee Babol University of Medical Sciences Babol Iran; ^2^ Student Research Committee Shiraz University of Medical Sciences Shiraz Iran; ^3^ Institute for the Developing Mind, Children's Hospital Los Angeles, Keck School of Medicine University of Southern California Los Angeles California USA; ^4^ Social Determinants of Health Research Center, Health Research Institute, Department of Biostatistics & Epidemiology, School of Public Health Babol University of Medical Sciences Babol Iran; ^5^ Infectious Diseases and Tropical Medicine Research Center, Health Research Institute, Department of Parasitology and Mycology Babol University of Medical Sciences Babol Iran; ^6^ Division of GI/Liver, Department of Medicine, Keck School of Medicine University of Southern California Los Angeles California USA

**Keywords:** Iran, mortality, neoplasms, trends

## Abstract

**Background:**

Cancer is a major cause of mortality. Timely information about cancer mortality trends is essential for prioritizing health programs.

**Aims:**

This study aims to use a Joint point regression model to predict the mortality rates of the top 10 common cancers in Babol City.

**Methods:**

This cross‐sectional study considered all registered cancer‐related deaths from 2013 to 2021 in the Babol University of Medical Sciences' death registration and classification system. Crude and age‐standardized rates and cancer trends were calculated and predicted for the next 5 years, overall and by type of cancer and gender.

**Results:**

Over these 9years, 2417 deaths from the 10 common cancers were recorded. We observed an increase in mortality rates with a slope of 12.05% from 2013 to 2016, and a gentler slope of 3.2% from 2016 to 2021. Cancer mortality rates are predicted to increase by 6.43% in the next 5 years without intervention. Detailed analysis indicates that breast cancer will have the highest mortality rate during 2022–2026, rising by 13.6% annually. Predictions based on gender indicate that, breast cancer mortality will increase by 13.6% annually for women. Also, stomach cancer mortality rates will increase by 0.15% in men annually.

**Conclusion:**

Cancer mortality in Babol remains a significant public health issue with an increasing trend. Nevertheless, these rising mortality rates require urgent interventions, including cancer prevention programs, increased access to medical services, and improved quality of life.

## 1 Introduction

Cancer, a leading cause of mortality globally, has seen a shift in mortality patterns, as reported by the World Health Organization [1, 2]. In 2020, the International Agency for Research on Cancer (IARC), affiliated with the WHO, revealed that there were 19.3 million new cancer cases and 10 million cancer‐related deaths, underscoring the global impact of this disease. The global burden of cancer is projected to increase by 47% by 2040 compared to 2020 [3]. In Iran, cancer mortality ranks third after cardiovascular diseases and accidents [4]. In 2019, Iran witnessed a mortality rate of 463.97 per 100 000 population for both sexes across all ages, with non‐communicable diseases accounting for 387.33 per 100 000, of which cancers comprised 79.23 deaths per 100 000 [5]. It is also anticipated that this trend, with cancer as the third leading cause of death, will persist until 2035 [6].

Mortality is the first demographic indicator to capture human attention more than any other demographic event, as all societal efforts aim to increase human lifespan. Countries' economic, social, and health policies have always been directed towards reducing mortality and increasing lifespan [7]. On the other hand, identifying and monitoring mortality trends is crucial for setting health priorities, allocating resources, and focusing on health‐oriented development in the healthcare sector [8]. Regression models for predicting cancer mortality, such as the join point regression, are widely used in epidemiological studies in medical sciences for modeling time trends of mortality and incidence data [9]. For instance, a study by Shaji et al. in India utilized this model to reveal an upward trend in mortality for several cancers, including lung, breast, and colorectal, from 2000 to 2019 [10]. Another investigation, covering the period from 1978 to 2009, showed that overall cancer mortality, especially for stomach and lung cancers, declined in Italy after the 1980s [11]. Additionally, various studies employing this model have explored cancer mortality trends across different timeframes [12, 13, 14], all emphasizing its significance in capturing trend changes and forecasting future patterns. Furthermore, changes in risk factor exposure or the introduction of new screening programs or other interventions might influence the trends in rates and frequencies. Similarly, sudden changes in population structure, like new migration patterns, might lead to trend shifts in the actual numbers of cancer cases. Therefore, in trend modeling over time using joinpoint analysis, it is vital to identify significant statistical trend changes [15].

Babol, located in northern Iran, and is the second‐largest city in the region with a population of over 500 000 and an area of approximately 15 781 km^2^. Situated in the coastal region of the Caspian Sea, this county benefits from a humid, temperate climate, and hosts a population that includes both urban and rural residents. Moreover, with its considerable healthcare infrastructure, the city has emerged as a major center for medical services and healthcare delivery in northern Iran [16]. Timely, join, and reliable information on the level and trends of mortality and its causes is fundamental for prioritizing health programs. The best source of data for mortality estimation, considered the gold standard, is the national vital events registration system, currently implemented in 30 provinces of the country, part of which includes the cancer mortality registration system [17]. Given the changes in the demographic and epidemiological profile of non‐communicable diseases and the need for planning and implementing effective interventions, this study aims to utilize a join point regression model to predict the mortality rates of the top 10 common cancers in Babol City over the study period. The Joinpoint regression model plays a vital role in cancer research due to its capacity to identify significant shifts in mortality trends and determine annual rates of change. This model simplifies complex analyses and accommodates irregular data, allowing researchers to detect critical changes in cancer mortality and evaluate the effectiveness of treatment and prevention interventions. Moreover, it provides a robust tool for predicting future trends and supporting strategic public health planning.

## 2 Method

The study population in this research comprises all recorded cancer‐related deaths in Babol City from 2013 to 2021, documented in the death registration and classification system of the Health Deputy of Babol University of Medical Sciences. The sampling method was a complete enumeration of registered deaths from the 10 most common cancers in the specified time frame. This project was approved by the Ethics Committee of Babol University of Medical Sciences with the code (IR.MUBABOL.HRI.REC.1402.036).

The sources of mortality causes are obtained from the Mortality Cause Registration and Classification System under the Health Deputy, based on reliable sources such as valid death certificates. These sources include cemeteries, forensic medicine, hospitals, and all trained physicians who receive a specialized booklet for recording causes of death from the Health Deputy. An important phase in the analysis of mortality data is the examination of the quality of information regarding causes of death. To this end, data were scrutinized for duplicate entries and cross‐checked against other registered information. Implausible cause of death codes regarding age and gender, lethal causes, and poorly defined or void codes were reviewed and corrected. Causes of death deemed impossible or extremely rare for a particular gender and age were queried and rectified by experts after consulting relevant records. Following the removal of duplicates, the redistribution of void and poorly defined codes was conducted. After validation by the Ministry of Health officials, the data attained the reliability for final reporting and citation in this study.

The causes of death were categorized using the 10th edition of the International Classification of Diseases and Mortality (ICD‐10). The codes for cancers included: C15 (esophagus), C16 (stomach), C32–34 (lung), C18–22 (colorectal), C50 (breast), C70–72 (brain, central nervous system), C61 (prostate), C22 (liver), C82–86, C96 (non‐Hodgkin lymphoma), and C25 (pancreas) [18].

This cross‐sectional study utilized join point regression analysis to predict the mortality of common cancers. Join point regression, a method where variables are divided into primary and independent categories, was employed. For each interval, a separate regression line is fitted, and the boundaries between segments are known as joinpoints. This analysis is a technique to examine significant points and the number of changes over a long period for populations and health trends. The term “joinpoints” indicates the start time of a change. join point regression creates statistically significant points relative to the previous point, forming segments and calculating an annual percentage change (APC) for each segment. Additionally, it provides the average annual percentage change (AAPC). Both measures were used to analyze the trend changes over these years. In this study, the response variable was cancer mortality, and the independent variable was the years under study (2013–2021).

Joinpoint is statistical software designed to analyze trends using models that consist of linear segments connected at ‘joinpoints,’ where the trend changes. Commonly used for cancer trend analysis, such as in NCI publications, it fits the simplest joinpoint model that the data supports, testing whether adding joinpoints improves the model's statistical significance. This is done using the Monte Carlo Permutation method, and the models can account for variations like Poisson distributions or logarithmic responses. Additionally, Joinpoint provides visual graphs for each model, from the minimum to maximum joinpoints, allowing users to clearly see trend changes [19].

For analysis aligned with the study objectives, Joinpoint software version 5.2.0 [20] was utilized. Based on the confirmed incidence cases through this software, the joinpoint time between two stages, the growth rate, the first stage (slow growth), and the second stage (rapid growth) were identified to demonstrate changing patterns. Also, the number and location of change points in the mortality trend were estimated. The joinpoint regression parameters were assessed using Lerman, Hudson, and permutation testing techniques. Both crude and age‐standardized rates and cancer trends were calculated and predicted for the next 5 years, overall and by type of cancer and gender. *p* values less than 0.05 were considered statistically significant.

## 3 Results

Over the 9 years of this study, a total of 2417 cancer‐related deaths were recorded in Babol City, with gastric cancer accounting for over one‐fifth of these cases. Approximately 60% of these cases were male (Table 1).

**TABLE 1:** Mortality cases of the 10 most common cancers in Babol City, 2013–2021.

Variable	Subgroup	*N* (%)
Years	2013	184 (7.6)
	2014	196 (8.1)
	2015	207 (8.6)
	2016	286 (11.8)
	2017	272 (11.3)
	2018	308 (12.7)
	2019	322 (13.3)
	2020	315 (13.0)
	2021	327 (13.5)
Cancers (ICD code)^a^	Stomach (C16)	538 (22.3)
	Lung (C32–34)	383 (15.8)
	Colorectum (C18–22)	284 (11.8)
	Breast (C50)	256 (10.6)
	Brain, central nervous system (C70–72)	228 (9.6)
	Prostate (C61)	213 (8.8)
	Liver (C22)	168 (7.0)
	Non‐Hodgkin lymphoma (C82–86, C96)	140 (5.3)
	Oesophagus (C15)	110 (4.6)
	Pancreas (C25)	105 (4.3)
Gender	Male	1440 (59.6)
	Female	977 (40.4)

^a^ ICD‐10 was used.

**FIGURE 1:**
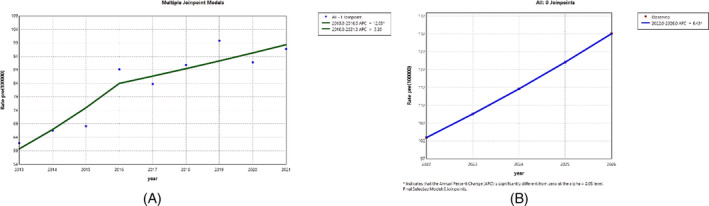
Trend and prediction of all cancers for all patients. (A) Trend from 2013 to 2021. (B) Prediction from 2022 to 2026.

Figure 1A illustrates the overall mortality trend based on the standard mortality rate (SMR) for the 10 most common cancers per 100 000 people in Babol from 2013 to 2021. This chart shows an increasing mortality trend with a slope of 12.05% from 2013 to 2016, followed by a more gradual slope of 3.2% from 2016 to 2021. Additionally, Figure 1B displays the predicted trend for the next 5 years for these common cancers based on the AAPC, indicating an upward trend of 6.43%. This figure suggests that without intervention, we can expect an annual increase of 6.43% in cancer mortality rates.

**FIGURE 2:**
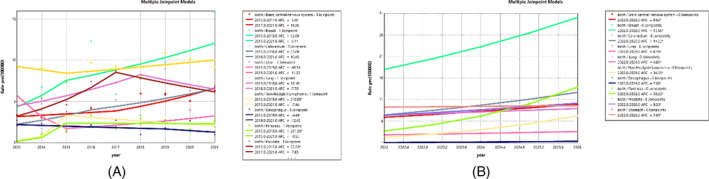
Trend and prediction of cancers for all patients. (A) Trend from 2013 to 2021. (B) Prediction from 2022 to 2026.

Figure 2A represents the mortality trend for the most common cancers, including brain, breast, colorectal, liver, esophageal, lung, prostate, lymph, and pancreatic cancers. Among the top 10 cancers over the years, breast cancer was at the forefront, witnessing an increase in mortality with a slope of 8.11% from 2014 to 2021. Following this was gastric cancer, with an APC of 2.95 from 2015 to 2021. Figure 2B examines the forecasted mortality trend for each of these cancers. The highest mortality rate over the next 5 years is projected for breast cancer, with an annual increase of 13.66% (AAPC), followed by pancreatic cancer, with an annual increase of 39.20% (AAPC).

**FIGURE 3:**
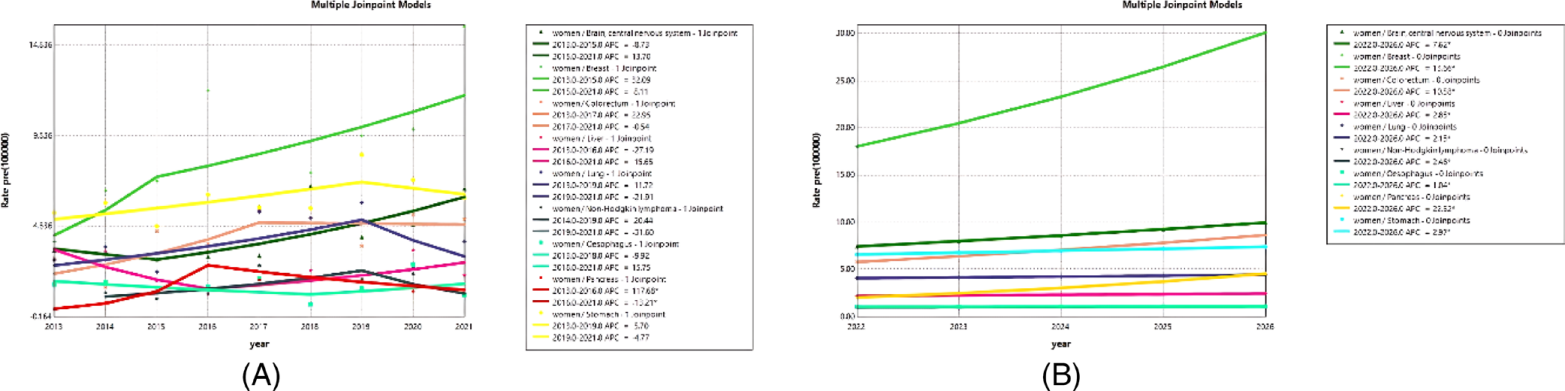
Trend and prediction of cancer for women. (A) Trend from 2013 to 2021. (B) Prediction from 2022 to 2026.

This study also analyzed mortality rates by gender and predicted trends for men and women. Figure 3A shows that breast cancer is the most common cancer among women, increasing from 2013 to 2015 with an APC of 32.09 and from 2015 to 2021 with an APC of 8.1. This was followed by gastric cancer, which had a high level and an increasing trend with an APC of 5.70 from 2013 to 2019. Breast cancer was observed with an AAPC of 13.6%, meaning that, without intervention, we can expect an average annual increase of 13.6% in breast cancer mortality among women (Figure 3B).

**FIGURE 4:**
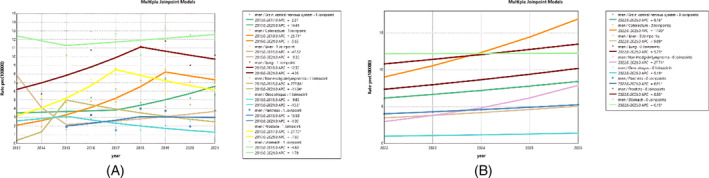
Trend and prediction of cancer for men. (A) Trend from 2013 to 2021. (B) Prediction from 2022 to 2026.

Figure 4 examines the mortality trend and 5‐year forecast for the most common cancers among men. The most common cancer in men was gastric cancer, which initially showed a slight decrease, followed by an increase in mortality. From 2015 to 2021, an APC of 1.79 was observed. Lung cancer, which initially showed a sharp increase followed by a decline, was next, with an APC of 12.32 from 2013 to 2018, followed by a slight decrease. In the male prediction phase, colorectal cancer is expected to increase annually by 17% (AAPC), and lung cancer, with an AAPC of 5.73%, follows colorectal cancer.

## 4 Discussion

This study examined the use of a join point regression model to predict the mortality rates of the most common cancers in Babol city during 2013–2021. The study's results show an increasing cancer mortality trend from 2013 to 2016 with a steep slope, followed by a gentler slope from 2016, consistent with global studies. High‐income countries (HICs) see decreasing specific cancer mortality rates, while low‐ and middle‐income countries (LMICs) experience increases. Factors such as smoking, obesity, and physical inactivity contribute to the rising rates in LMICs [21]. A study by Jung et al. predicts a continuing increase in the cancer burden in Korea, similar to our findings [22]. They indicated that, according to projections for 2015, lung, liver, and colorectal cancers would rank as the three most prevalent cancers in both men and women. In a related study conducted in Spain, Roura et al., examining data from 2005 to 2020, found that 1‐year mortality rates for stomach and lung cancers were the highest across both genders [23]. With an aging population, the cancer burden, particularly breast cancer, is expected to rise in Iran [24]. A study on breast cancer in Iran reported a significant tripling in age‐specific incidence nationally and locally, with a doubling in provincial disparities. Though national and local mortality from breast cancer has slightly decreased since 2000, positive annual mortality percentage changes in certain age groups indicate increasing years of life lost (YLLs) [25]. Another study by Jani et al. analyzing breast cancer trends in Europe between 2001 and 2017 found that, apart from Croatia, France, and Poland, most countries saw an increase in breast cancer mortality. They also highlighted that Denmark showed the greatest reduction in mortality, with an APC of −3.2% [26]. Contrary to the downward trend observed in most European countries, our study's findings differed. The reduction in breast cancer mortality across much of Europe could be attributed to better access to healthcare and welfare services. However, our findings on pancreatic cancer present a different picture. This study showing a significant increasing trend in pancreatic cancer. One study reported a minor decrease in age‐standardized mortality rates of pancreatic cancer from 1999 to 2004 [27], while another showed a continual increase in incidence and mortality rates with age in Iran [28], with men being more affected. Additionally, Llic et al. reported that since 1990, pancreatic cancer mortality has increased in all countries except for Canada and Mexico [2]. The observed reduction in these two countries, unlike in others including Iran, could be due to advancements in screening practices and early treatment interventions. Also, risk factors include tobacco use, aging, and lifestyle changes. Due to the lack of early symptoms or specific markers for early diagnosis of pancreatic cancer, it i s often identified late, leading to low survival rates [28].

The study's findings indicate that gastric cancer is the leading cause of cancer mortality in Babol, aligning with previous research [29]. Gastric cancer appears more prevalent in northern Iran, including Babol, possibly due to differences in environmental factors such as diet and *Helicobacter pylori* infection [30]. A global study by Lin et al. predicted that by 2035, most countries, including China (APC for men: −3.2%), South Korea (APC for women: −6.2%), Canada (APC for men: −3.6%), and the United States (APC for women: −1.6%), will observe a decline in stomach cancer mortality for both sexes. However, countries like Thailand are expected to experience rising mortality rates for both men (APC: 3.5%) and women (APC: 4.7%) [31]. Although many nations are projected to see a decrease in stomach cancer mortality by 2035, factors such as population growth and aging may result in a higher absolute number of deaths in some areas. Countries with lower economic resources or limited healthcare access may continue to face increasing mortality rates, particularly among younger populations. Also, the dietary habits in northern Iran, particularly the consumption of salty foods, could be risk factors for increased *Helicobacter pylori* infection, leading to higher gastric cancer mortality. Additionally, our study finds higher cancer mortality in men than in women. Studies suggest women have lower mortality rates due to greater engagement with healthcare services, leading to earlier screening, diagnosis, and treatment of diseases. Lifestyle factors like smoking, alcohol consumption, and unhealthy dietary patterns are more prevalent among men [32, 33]. In exploring the underlying mechanisms, carcinogens in cigarette smoke are known to cause cancer by inducing DNA damage and increasing inflammation in lung and other tissues. Prolonged smoking also weakens the immune system, making it more difficult to inhibit the spread of cancer [34, 35]. In terms of diet, the consumption of saturated fats contributes to inflammation and metabolic disruptions, potentially increasing the risk of gastrointestinal and breast cancers. Additionally, inadequate intake of fruits and vegetables, which are rich in antioxidants, diminishes the body's capacity to counter oxidative stress, heightening the likelihood of cancer [36]. Environmental factors, such as poor water quality and pollution, particularly in rural areas dependent on surface or well water, may further predispose individuals to gastrointestinal cancers. High salt consumption and the frequent intake of salt‐preserved processed foods also pose significant risks for stomach cancer. The common use of such foods in this region can lead to gastric ulcers, which may eventually develop into cancer [37, 38].

Moreover, our study shows that the most common cancer mortality in women is from breast and gastric cancers. At the same time, its stomach, lung, and colorectal cancers in men, aligning with another study's findings [39]. The mortality rate from gastric cancer is high in East and Central Asia and Latin America. Afghanistan, Oman, Sudan, and Yemen, despite mortality reductions from 1990 to 2017, have the highest rates [40]. *Helicobacter pylori* infection, diet, and lifestyle factors account for 33%–50% of gastric cancers, with significantly lower rates in women than men [40, 41]. Lung cancer, as the second leading cause of cancer mortality in men in our study, is complex but relates to aging, population growth, and changes in the prevalence and distribution of risk factors [42]. Smoking is the leading risk factor, accounting for over 90% of lung cancer cases. Approximately 80% of smokers worldwide live in low‐ and middle‐income countries [42, 43]. Colorectal cancer (CRC), as in this study, is the third leading cause of cancer mortality in men globally [44]. For women, breast cancer is the primary cause of cancer‐related death in this study and globally. In the Eastern Mediterranean Region (EMR), including Iran, breast cancer has the highest incidence and mortality rates among women [45, 46].

## 5 Limitations and Strengths

Our study used only age and gender as variables for predicting mortality rates. Future studies could improve accuracy by incorporating other variables like marital status, socioeconomic status, and family history of cancer. Moreover, based on our data, conducting a competing risk analysis was not feasible. Another limitation is that this study focused solely on Babol City. A geographic distribution study of cancers in different areas of the city or across Iran could provide more insights into the risk factors in these regions. However, our study employed a join point regression model, a valid statistical model for mortality prediction, especially with few data points. We aimed to provide comprehensive and helpful information about the cancer situation in Babol.

## 6 Conclusion

The study reveals that gastric cancer is the most prevalent in Babol, with most cases in men. The overall standardized mortality trend increased steeply until 2016 then rose slowly. Breast cancer is expected to have the highest mortality rate in the next 5 years, with pancreatic cancer also showing a significant increase. The most common cancers in women are breast and stomach, and in men, they are stomach, lung, and colorectal. These results indicate an increasing trend in cancer mortality in Babol, posing significant public health concerns. Addressing this issue requires actions from health policymakers and professionals, including cancer prevention programs, improved access to cancer diagnosis and treatment services, and enhancing the quality of life for cancer patients.

## Author Contributions

P.E. and S.K. collected the data set by H.N. and M.K. P.E., S.K., and M.V. drafting the initial structure of the manuscript and H.N., S.D., S.K., and L.S. editing it. All authors wrote or supervised the content of the manuscript and approved the final manuscript.

## Ethics Statement

The Helsinki Declaration was followed for all methods. Babol University of Medical Sciences' Ethics Committee reviewed and approved the study (IR.MUBABOL.HRI.REC.1402.036), including the waiver of informed consent.

## Conflicts of Interest

The authors declare no conflicts of interest.

## Data Availability

Upon reasonable request, the datasets used in the current study can be obtained from the corresponding author.
